# Coagulation and Transfusion Informatics in Chronic Liver Disease: A Data Linkage Study of Emergency Department Presentations

**DOI:** 10.1002/jha2.70101

**Published:** 2025-07-10

**Authors:** Akmez Latona, Emma Smith, James Grant, Biswadev Mitra

**Affiliations:** ^1^ Emergency Department Ipswich Hospital Ipswich Queensland Australia; ^2^ Statewide Anticoagulation Committee, Queensland Health Brisbane Queensland Australia; ^3^ LifeFlight Retrieval Medicine Toowoomba Queensland Australia; ^4^ School of Public Health and Preventive Medicine Monash University Melbourne Victoria Australia; ^5^ The University of Queensland Faculty of Medicine Herston Queensland Australia; ^6^ Data Analytics, Digital Services West Moreton Health Ipswich Queensland Australia; ^7^ Office of the Chief Clinical Information Office eHealth Queensland Brisbane Queensland Australia; ^8^ Emergency & Trauma Centre, Alfred Health Melbourne Victoria Australia; ^9^ Expeditionary Health Squadron, Royal Australian Air Force Amberley Queensland Australia

**Keywords:** bleeding, coagulation, data linkage, Emergency Department, liver disease, transfusion

## Abstract

**Objective:**

To describe the application of data linkage for analysing coagulation abnormalities and blood transfusion practices in patients with chronic liver disease (CLD) presenting to emergency departments (EDs).

**Methods:**

Patients with CLD presenting to 104 Queensland Health EDs (January 2016–August 2023) were identified using International Classification of Diseases codes. Phase 1 deterministically linked ED, admission, pathology, transfusion and death records using unique identifiers. Phase 2 used Structured Query Language (SQL) to capture transfusion timing. The model incorporated data from both digital and non‐digitalised hospitals.

**Results:**

Phase 1 linkage identified 36,643 ED presentations, 443,367 admissions, 47,357 deaths, 3,004,236 pathology results and 140,687 blood transfusion events. Phase 2 identified 23,578 ED presentations by 11,961 patients, linked to 20,312 admissions, 921 deaths, 22,284 full blood counts (FBC), 19,408 coagulation profiles, 3068 blood gases, 457 rotational thromboelastometry (ROTEM) and 53 thromboelastography tests. Transfusion data were linked to 1616 presentations, including of 1358 red blood cell (RBC) transfusion episodes, 330 fresh frozen plasma, 324 cryoprecipitate, 418 platelets, 51 fibrinogen concentrate and 280 Prothrombinex‐VF administration episodes. High linkage rates were achieved for FBC (99.4%), coagulation profile (97.6%) and biochemistry (92.3%), while linkages for blood gas (34.6%), ROTEM (13.8%) and thromboelastography (2.5%) were less frequent. Massive transfusions occurred in 27 presentations (≥ 4 RBC units in 4 h) and in 22 presentations (≥ 10 RBC units in 24 h), with 100% linkage for FBC and coagulation profiles in both groups.

**Conclusion:**

The feasibility of data linkage to investigate coagulation abnormalities and transfusion in CLD patients was demonstrated. This model provides a scalable method for haemovigilance and transfusion research.

**Trial registration:**

The authors have confirmed clinical trial registration is not needed for this submission.

## Graphical abstract

This study demonstrates the feasibility of applying a data linkage framework to evaluate coagulation abnormalities and transfusion practices in patients with chronic liver disease (CLD) presenting to emergency departments (EDs). Coagulation and transfusion data are typically fragmented across hospitals and information systems, limiting comprehensive analysis at a statewide or national level. This study addresses that gap by integrating multiple datasets and outlining the linkage challenges encountered in applied haematology. Using statewide data, we showed that conventional coagulation profiles and viscoelastic haemostatic assays (ROTEM and TEG) can be linked to transfusion events, including at defined intervals from ED arrival—within 4 h, within 24 h, and in relation to massive transfusion thresholds. This approach enables temporal alignment of laboratory results with transfusion timing, supporting real‐world evaluation of clinical decision‐making during early haemostatic resuscitation. It includes mapping test timing, ED presentation and blood component use across all hospitals, addressing the longstanding fragmentation of transfusion and coagulation records. The resulting dataset provides a foundation for further analyses examining associations between coagulation parameters and transfusion decisions. The methodology offers a scalable informatics model for haemovigilance, with broader applicability to statewide or national transfusion‐focused initiatives, including audit, research and surveillance programs.

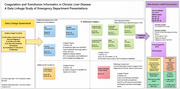

## 1 Introduction

Chronic liver disease (CLD) poses a significant global healthcare burden, with patients frequently presenting to emergency departments (EDs) with coagulation abnormalities and bleeding [1]. These patients often require blood transfusions for haemostasis and to restore oxygen‐carrying capacity [2]. However, blood is a precious, donated resource. While one in three Australians requires a blood transfusion in their lifetime, only one in 30 donates blood, resulting in a chronic mismatch of supply and demand [3]. Despite the critical role of blood transfusion in the care of patients with CLD and the paramount importance of judicious use of blood components, much of the relevant data on clinical characteristics, pathology, blood component use and patient outcomes are fragmented across different hospital systems, limiting assessments of patient blood management [4].

Data linkage integrates multiple datasets to construct a comprehensive view of a patient's healthcare journey [5]. Identifiers, such as name and date of birth, are used solely for linkage and excluded from final datasets. To protect privacy, project‐specific identifiers enable individual‐level analysis without revealing personal identities [6]. This approach is widely adopted across health systems. The United Kingdom's Clinical Practice Research Datalink connects primary care with hospital, cancer and mortality records [7]. Canada's Institute for Clinical Evaluative Sciences links administrative datasets for chronic disease research [8]. Australia's Centre for Data Linkage enables access to emergency, hospital, cancer and perinatal records [9]. Within transfusion medicine, pathology and transfusion data have previously been linked. Patterson et al. identified red blood cell (RBC) transfusions among women giving birth in New South Wales [10]. Palfy et al. examined blood product use in South Australia [11]. Wiggins et al. incorporated pathology data to study chronic kidney disease in Tasmania [12]. However, integration of coagulation studies with transfusion records remains limited. In CLD, data linkage has addressed aetiologies, mortality and health disparities but has yet to combine coagulation studies with transfusion data [13, 14].

Data linkage for investigating coagulation abnormalities and blood transfusion practices in ED presentations remains unexplored [15]. The aim of this study was to evaluate the feasibility of data linkage in a statewide cohort of patients with CLD. This clinician‐driven approach was aimed at establishing the groundwork for future research in applied haematology in CLD at a statewide level, with potential implications for haemovigilance practices.

## 2 Methodology

### 2.1 Study Design and Data Sources

This proof‐of‐concept study utilised multiple datasets from Queensland Health (QH), capturing all presentations to the 104 EDs across Queensland's public hospital system. While QH is progressing towards full digitalisation of its hospitals, some facilities currently use the integrated electronic medical record (ieMR), while others rely on a combination of paper‐based processes and standalone digital systems such as Emergency Department Information System (EDIS). This study used data extracted from centralised databases, ensuring statewide consistency across both digital and non‐digital hospitals. The datasets linked included the Emergency Data Collection (EDC), Queensland Health Admitted Patient Data Collection (QHAPDC), Queensland Health Laboratory Information System, AUSLAB and the Death Registry (DR).

Queensland's EDC provides demographic, administrative and clinical data, including variables such as presentation date and time, length of stay (LOS), Australasian Triage Scale, hospital and health service (HHS), diagnosis codes (principal and additional diagnoses), age group, biological sex, Aboriginal or Torres Strait Islander status, mortality and ED departure status. QHAPDC captures hospital admission details, including diagnosis (primary and other diagnoses), ICU admission, ICU LOS, hospital LOS, procedures and discharge status. Queensland's DR contains death dates, age at death and ICD‐coded cause of death. AUSLAB, Pathology Queensland's platform, provides data on pathology results, including coagulation parameters and blood transfusions. A transfusion event was defined as the release of an individual blood product, with each unit recorded as a separate entry in AUSLAB. Only completed transfusions were included; products that were dispatched but returned to the laboratory without administration were excluded by Pathology Queensland prior to the release of the transfusion dataset. Further details on these databases are available on the Data Linkage Queensland (DLQ) [6].

### 2.2 Cohort Selection

The study cohort included adult patients (≥ 18 years) with a diagnosis of liver disease who presented to QH EDs or were admitted to QH hospitals between 1 January 2016 and 31 August 2023. Patients were identified using ICD‐10‐AM (International Classification of Diseases, 10th Revision, Australian Modification) codes relevant to liver disease (Appendix 1), searched in both the EDC (for principal or additional diagnoses) and QHAPDC (for primary or other diagnoses).

### 2.3 Data Linkage Process

**FIGURE 1:**
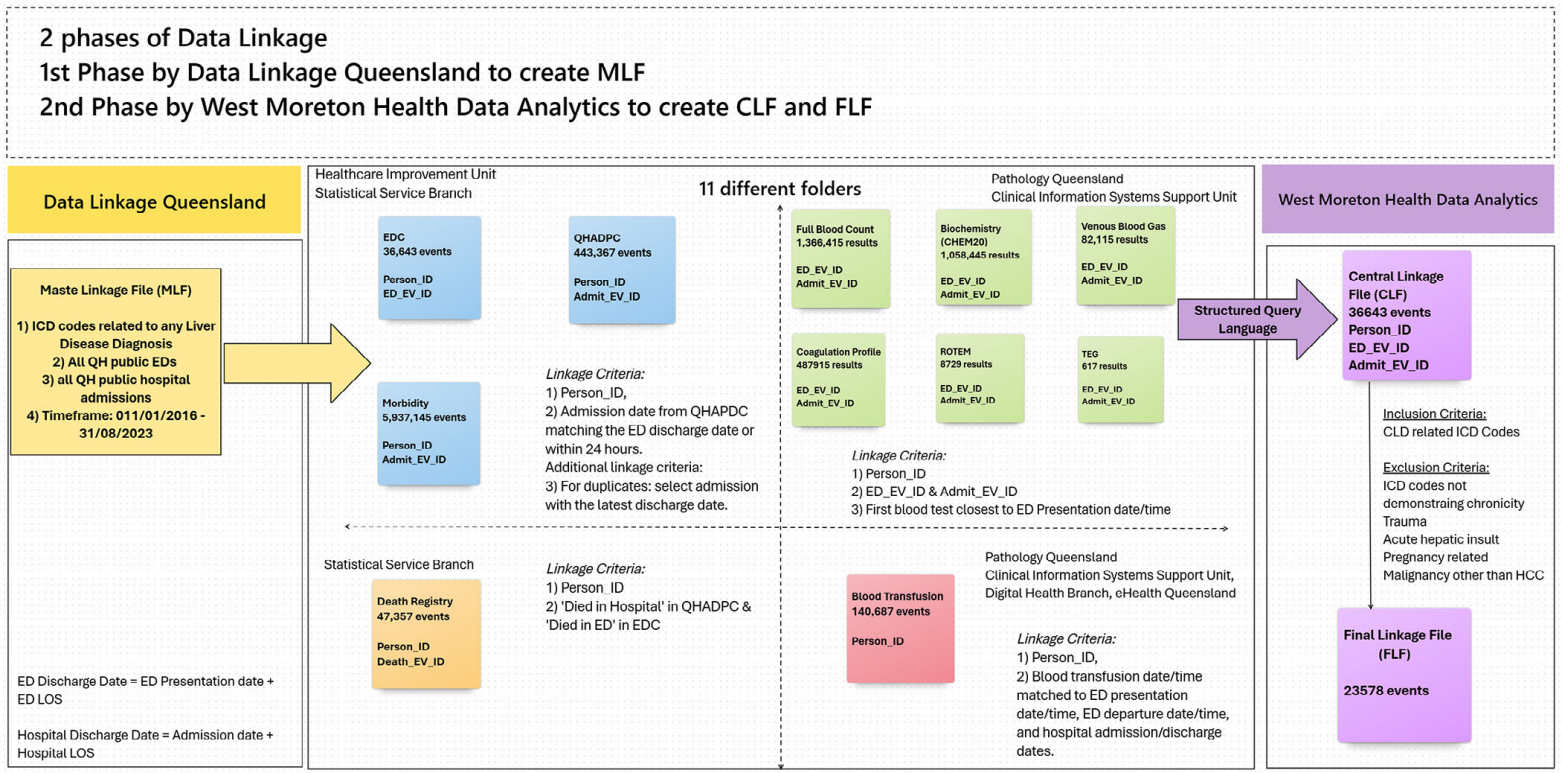
Shows databases and methodology of Phase 1 data linkage. Chem20, biochemistry; CLD, chronic liver disease; EDC, emergency data collection; FBC, full blood count; FFP, fresh frozen plasma; ICD code, International Classification of Diseases, tenth revision, clinical modification; QHADPC, Queensland Hospital admitted patient data collection; RBC, red blood cell; ROTEM, rotational thromboelastometry; TEG, thromboelastography. The ED discharge date was calculated by adding the ED presentation date/time to the ED LOS. Hospital discharge date from the admission was calculated by adding the admission date to the hospital LOS.

The data linkage process, illustrated in Figure 1, was conducted in two phases. In Phase 1, deterministic linkage was performed by DLQ using diagnostic codes from EDC and QHADPC to identify relevant patients. DLQ also linked death records from DR and pathology and transfusion data from AUSLAB through the Clinical Information Systems Support Unit (CISSU). A Master Linkage File (MLF) was created with a unique Person_ID linking all datasets [16]. De‐identified records were extracted into 11 files: EDC, QHADPC, full blood count (FBC), biochemistry (Chem20), venous blood gas (VBG), coagulation profile, rotational thromboelastometry (ROTEM), thromboelastography (TEG), blood transfusion, morbidity and DR.

The second phase of data linkage combined the 11 files to map a patient's journey from ED presentation to hospital admission, discharge or death. This included the first set of blood results on ED arrival and blood transfusion details at different timepoints, creating a single Central Linkage File (CLF). Inclusion and exclusion criteria were applied to the CLF to identify patients with CLD, using ICD codes indicative of CLD and excluding codes for trauma, acute hepatic insults and non‐chronic liver conditions. This process produced the FLF (Final Linkage File). Relevant ICD codes are provided in the appendix.

### 2.4 Linkage Criteria

The linkage process connected datasets using a combination of Person_ID, ED_EV_ID, Admit_EV_ID, date and time of ED presentation, LOS, admission and discharge dates, pathology results and blood transfusion data. Figure 1 outlines the specific linkage criteria for each database. Linkage was performed at both the person and event levels. Person‐level linkage used the unique patient identifier (Person_ID), which was consistent across all datasets. Event‐level linkage relied on EV_IDs: ED_EV_ID for ED presentations and Admit_EV_ID for hospital admissions, uniquely identifying each event. While EV_IDs linked morbidity data to QHAPDC admissions, they did not directly connect EDC, QHAPDC and DR datasets. Facility_ID, which identifies QH facilities, was excluded to capture patients transferred from the ED to another hospital for admission. Time‐based criteria aligned presentation, admission and discharge times to ensure continuity of the patient journey. For cases with multiple admissions linked to a single ED event (e.g., inter‐hospital transfers), the admission with the latest discharge date was retained.

### 2.5 Outcomes

**FIGURE 2:**
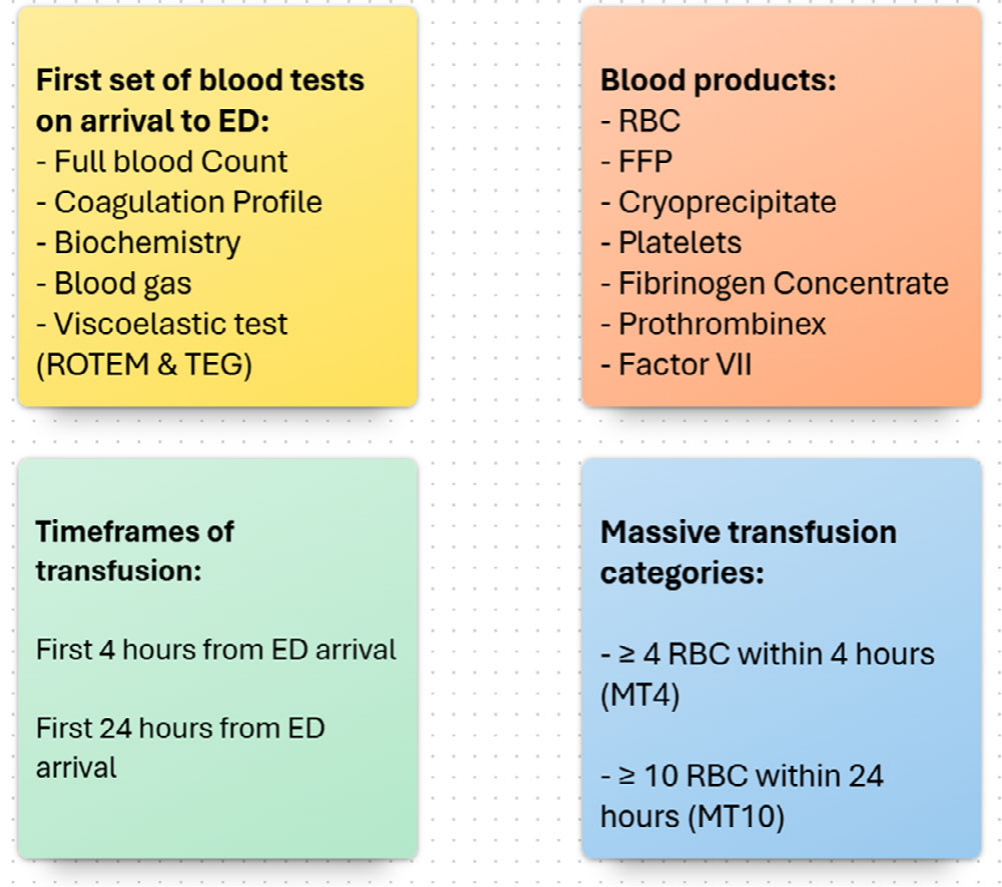
Shows the categorisation of pathology results and blood transfusion. FFP, fresh frozen plasma; RBC, red blood cell; ROTEM, rotational thromboelastometry; TEG, thromboelastography.

Figure 2 illustrates the outcomes. The first set of blood tests linked to ED arrival, including FBC, Chem20, coagulation profile, blood gas and viscoelastic tests, were recorded. Linked blood product types included RBC, fresh frozen plasma (FFP), cryoprecipitate, platelets, fibrinogen concentrate, Prothrombinex‐VF and factor VII, assessed at two timeframes: within the first 4 h and within the first 24 h of ED arrival. Massive transfusion (MT) linked to ED presentations was also recorded, with MT defined as ≥ 4 RBC units within 4 h (MT4) or ≥ 10 RBC units within 24 h (MT10). The proportions of ED presentations with linked pathology results, linked blood component transfusions and MTs were documented. Additionally, the proportion of linked pathology results among patients who received transfusions was recorded.

### 2.6 Data Analysis

Data were processed using SQL to link patient records across datasets. Left join syntax ensured all ED presentations from EDC were retained as the base, enriched with data from QHADPC, DR, morbidity, pathology and blood product datasets using linkage identifiers. Ethical approval was obtained from Metro South Human Research Ethics Committee (HREC/2023/QMS/102288).

## 3 Results

### 3.1 Phase 1: Initial Data Linkage (MLF)

Phase 1 involved data from 104 EDs, yielding 36,643 ED presentation episodes in the EDC, 443,367 admission episodes in QHADPC, 5,937,145 rows of ICD codes in the morbidity file and 47,357 deaths in DR. Pathology data extracted included 1,366,415 FBC results, 1,058,445 Chem20 results, 487,915 coagulation profiles, 82,115 blood gas results, 8729 ROTEMs and 617 TEGs. A total of 140,687 blood transfusion events were recorded (Figure 1).

### 3.2 Phase 2: Data Linkage (CLF)

**FIGURE 3:**
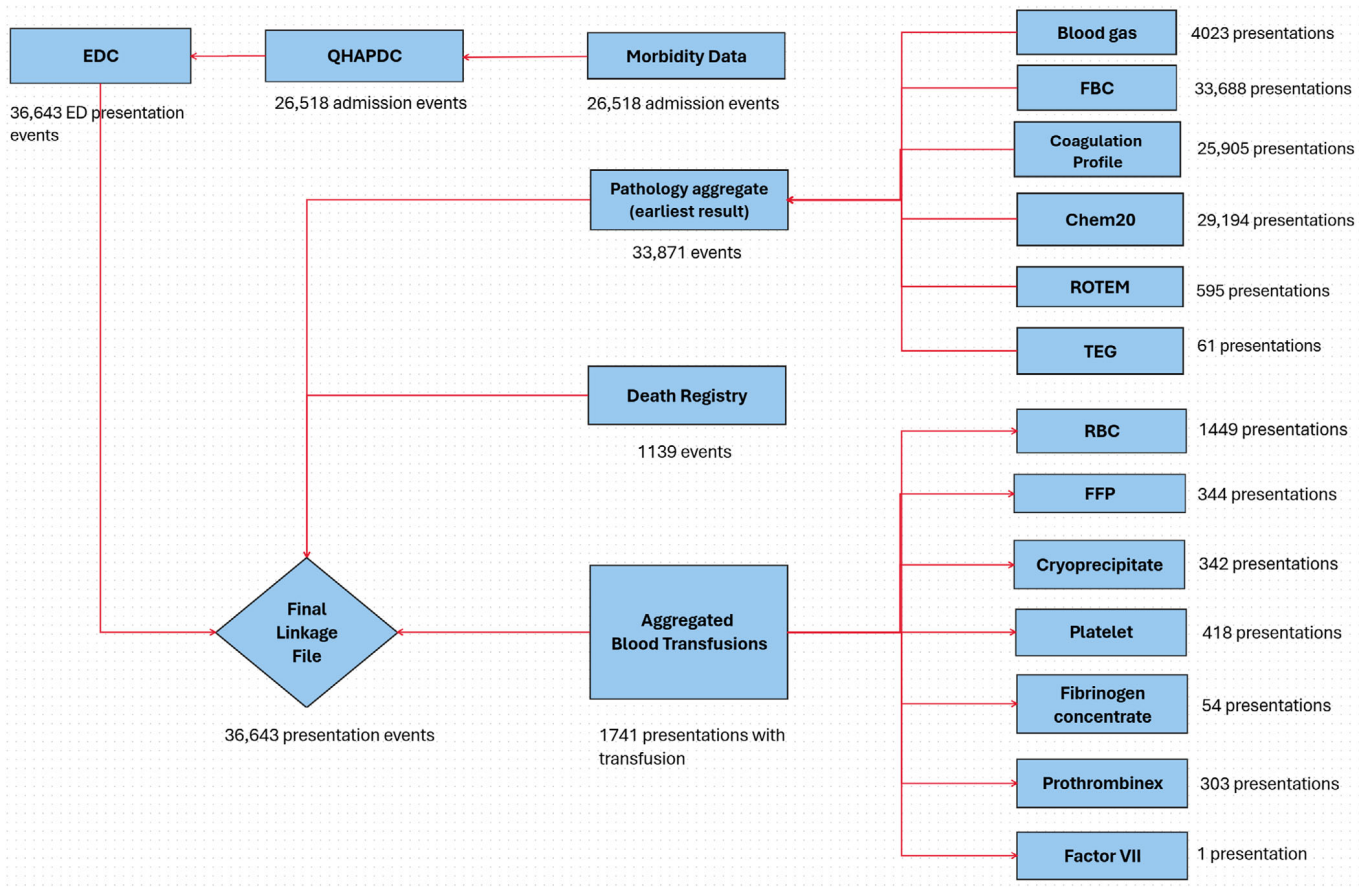
Shows the Phase 2 linkage by West Moreton Health Data Analytics. Chem20, Biochemistry; EDC, Emergency Data Collection; FBC, Full blood count; FFP, Fresh frozen plasma; QHADPC, Queensland Hospital Admitted Patient Data Collection; RBC, Red blood cell; ROTEM, Rotational thromboelastometry; TEG, Thromboelastography.

In Phase 2, West Moreton Health Data Analytics integrated 36643 ED presentations across 11 databases, representing 21,842 unique patients, using a left join on EDC (Figure 3). The resulting CLF comprises 36,643 ED events linked to 26,518 hospital admission events in QHAPDC, with all admissions successfully linked to morbidity records. A total of 33,871 pathology records, representing the earliest blood tests on hospital arrival, were identified. These included 33,688 FBC, 29,194 Chem20, 25,905 coagulation profiles, 4023 blood gas, 595 ROTEM and 61 TEG records.

### 3.3 Phase 2: FLF

**FIGURE 4:**
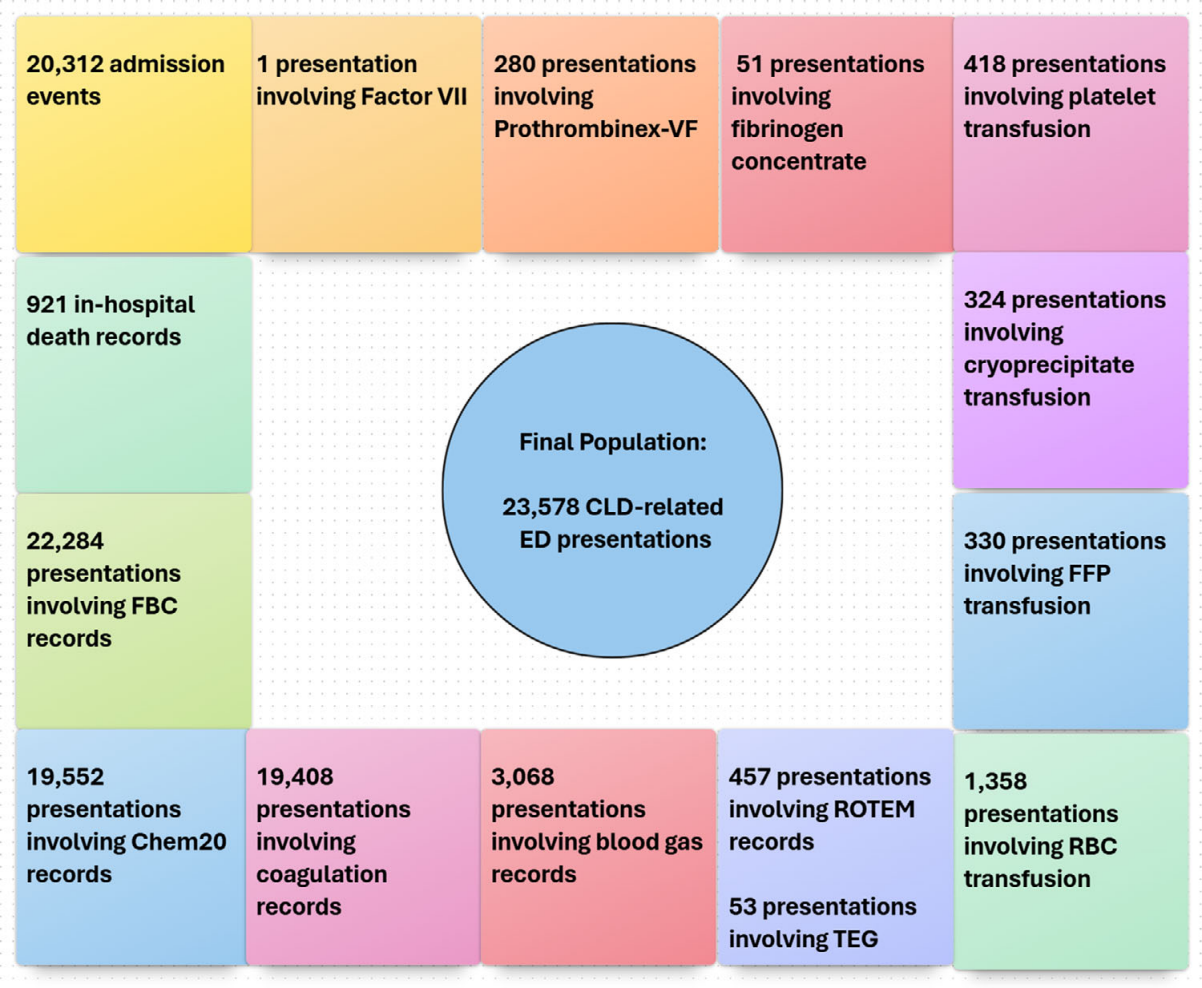
Shows the final population: 23578 CLD‐related ED presentations, linked to admission, pathology and transfusion records. Chem20, Biochemistry; FBC, Full Blood Count; FFP, Fresh frozen plasma; RBC, Red blood cell; ROTEM, Rotational thromboelastometry; TEG, Thromboelastography.

After applying inclusion criteria for CLD, the FLF included 23,578 CLD‐related ED presentations by 11,961 patients, linked to 20,312 admission events and 921 in‐hospital deaths (Figure 4). Earliest blood results linked to ED presentations included 22,284 FBC, 19,522 Chem20, 19,408 coagulation profiles, 3068 blood gas, 457 ROTEM and 53 TEG records. Blood transfusion data were linked to 1616 presentations, with specific transfusion types as follows: RBC (1358 events), FFP (330), cryoprecipitate (324), platelets (418), fibrinogen concentrate (51), Prothrombinex (280) and Factor VII (1).

Table 1 shows the results for transfusion‐linked presentations (*n* = 1616). Among these, 1607 FBC (99.4%), 1578 coagulation profiles (97.6%), 1491 Chem20 (92.3%), 549 blood gas (34.6%), 223 ROTEM (13.8%) and 40 TEG (2.5%) records were linked. For transfusions administered within the first 4 h (*n* = 272), linked pathology tests included 269 FBC (98.9%), 265 coagulation profiles (97.4%), 247 Chem20 (90.8%), 78 blood gas (28.7%), 86 ROTEM (13.2%) and 10 TEG (3.7%). Additionally, 27 patients received MT4, with 100% linkage to both FBC and coagulation profiles. For transfusions within the first 24 h (*n* = 908), linked tests included 901 FBC (99.2%), 882 coagulation profiles (97.1%), 829 Chem20 (91.3%), 263 blood gas (29.0%), 169 ROTEM (18.6%) and 26 TEG (2.9%). Notably, 22 patients were managed with MT10, with FBC and coagulation profile linkage in 100% of cases. There were 921 in‐hospital death records linked to EDC and QHAPDC events.

**TABLE 1:** Linkage results in patients who received blood transfusions.

Pathology test	Linked results	% Linkage
**Overall blood transfusion patients (*n* = 1616)**		
FBC	1607	99.4%
Coagulation profile	1578	97.6%
Chem20	1491	92.3%
Blood gas	559	34.6%
ROTEM	223	13.8%
TEG	40	2.5%
**Blood transfusions in the first 4 h (*n* = 272)**		
FBC	269	98.9%
Coagulation profile	265	97.4%
Chem20	247	90.8%
Blood gas	78	28.7%
ROTEM	86	13.2%
TEG	10	3.7%
**Massive transfusion (≥ 4 RBC units in first 4 h, *n* = 27)**		
FBC	27	100%
Coagulation profile	27	100%
**Blood transfusions in the first 24 h (*n* = 908)**		
FBC	901	99.2%
Coagulation profile	882	97.1%
Chem20	829	91.3%
Blood gas	263	29.0%
ROTEM	169	18.6%
TEG	26	2.9%
**Massive transfusion (≥ 10 RBC units in first 24 h, *n* = 22)**		
FBC	22	100%
Coagulation profile	22	100%

Abbreviations: Chem20, biochemistry; FBC, full blood count; RBC, red blood cells; ROTEM, rotational thromboelastometry; TEG, thromboelastography.

## 4 Discussion

There is an increasing number of ED presentations related to coagulopathy and bleeding in patients with CLD [17]. With data on coagulation and blood transfusion segregated across both individual HHS and different databases, comprehensive analysis of patient blood management has been limited [18]. While several digital technologies have been developed to optimise hepatology care and address healthcare disparities, none specifically target coagulation or blood transfusion [19]. This study is the first to utilise data linkage across all ED presentations in a statewide population to analyse coagulation and blood transfusion practices, establishing a novel framework for applied haematology and haemovigilance at a statewide level.

In the initial phase, we included ICD codes for all types of liver disease to capture a broader, more comprehensive patient range and to evaluate which conditions should be prioritised. After completing the data linkage, we refined our inclusion criteria to focus specifically on patients with CLD, characterised by coagulation abnormalities associated with rebalanced haemostasis [20]. Although a more targeted approach—requesting ICD codes for CLD upfront during the master linkage—might have been more efficient, the broader initial scope confirmed that blood transfusions primarily occurred in presentations associated with CLD‐ICD codes, rather than non‐CLD ICD liver codes. This informed and justified our decision to narrow the focus to the CLD cohort for more detailed analysis.

In this study, inclusion criteria for the CLD cohort were broadened to include ICD codes for both cirrhosis and chronicity to capture diagnosed and undiagnosed cirrhosis—particularly in rural and remote areas where access to timely hepatology care for formal diagnosis may be limited. To minimise the inclusion of patients whose presentation was unrelated to CLD, emergency department presentation data (EDC) were used. In EDC, diagnosis coding reflects the primary reason for the ED presentation; patients with a history of CLD are not coded as such unless it is clinically relevant to the acute presentation. This contrasts with QHAPDC admission data, where historical diagnoses may be included regardless of relevance to the current episode. This approach better captures presentations and complications acutely related to CLD. It aligns with the most specific algorithm described by King et al. and was selected to ensure consistency in the analysis of coagulation abnormalities and transfusion practices in future work by this group [21].

Linkage of pathology results to transfusion episodes enables assessment of the indications and effects of transfusion [22]. By characterising blood transfusion into different component types and MTs, this dataset will enable investigations into transfusion practices among patients managed with larger volumes of blood components and those with critical bleeding [23]. Categorising patients by health services will highlight regional variations and have implications for equitable blood resource planning. The success of this data linkage demonstrates its feasibility and opens the door to other large‐scale analyses in other clinical domains, such as trauma and other types of medical coagulopathy. Initially, Facility_ID was used to trace patients from ED presentation to hospital admission. However, this approach missed 826 cases involving transfers to different hospitals, preventing linkage to admission records. Removing Facility_ID as a linkage variable improved the capture of these admissions.

This comprehensive dataset revealed several challenges. Initially, linkage between EDC and QHADPC relied on Person_ID and admissions within 24 h of ED discharge. However, duplications occurred when patients were transferred between hospitals within 24 h, resulting in multiple admissions linked to one ED event. To address this, we retained the admission with the later discharge date, as it typically reflects the definitive care episode, ensuring accurate representation of clinical details, including comorbidities, mortality and ICD codes. This approach minimised duplications and improved data integrity by focusing on the patient's primary treatment episode. Another complexity was linking the 487,915 coagulation profile results, which included all patients presenting to the ED and all hospital admissions, regardless of whether they were admitted via the ED. To specifically link ED presentations and subsequent admissions from the ED, we used Person_ID and the date/time of coagulation tests within the ED timeframe, yielding 81,850 matches. Incorporating event_ID improved accuracy to 87,513 matches. The discrepancy was likely due to patients leaving the ED before their blood samples were processed in the laboratory, often because of short ED stays or rapid transfers, such as those to the operating theatre. To ensure maximum accuracy, both Person_ID and event_ID were used for the final linkage. Unlike previous studies focused on inpatient data, our study uniquely uses ED data as the initial linkage point, accounting for both admissions and inter‐hospital transfers, thereby addressing complexities specific to ED presentations [24].

The AUSLAB database includes a dedicated transfusion tab that records whether blood products were dispatched, returned or wasted, allowing clear identification of transfusions that were completed and administered to patients. For this study, we requested and obtained only transfusion events that were completed and administered. Historically, transfusion data have been studied at the HHS level, with smaller sample sizes limiting statistical power and risk factor adjustment [25]. Our statewide data linkage includes 140,687 recorded transfusion events for all liver diseases, representing 5% of patients linked to transfusion events, and provides a substantially large sample size. A comparative linkage was reported by Patterson et al., linking four administrative databases in New South Wales to identify 4642 (2%) RBC transfusions among 425,036 patients. Their study faced limitations due to staggered hospital participation and reliance on data submissions to central repositories [10]. Similarly, Palfy et al. analysed South Australia's blood product usage between January 2018 and June 2019, reporting 143,192 transfusion records (38% excluded) and 7,897,451 blood test records, achieving a 62.3% linkage rate for pathology tests [11]. Unlike these approaches, our methodology does not rely on hospital compliance for data submissions and successfully links a broader range of tests, including coagulation profiles, viscoelastic tests and blood gas results.

Wiggins et al. linked pathology results in Tasmania (2004–2017) from multiple providers, reducing 3.9 million records to just over 520,000 individuals through exact‐match de‐duplication [12]. In contrast, our study focused on a different approach, considering only the first blood test upon ED arrival, reducing 3,004,236 pathology results from Phase 1 of data linkage to 33,871 results linked to ED presentations. As expected, most, if not all, patients within the inclusion criteria who received blood transfusions would have been investigated with FBCs (99.4%) and a coagulation profile (97.6%) and thus provide a measure of the completeness of the linkage. The small proportion of patients without linked coagulation profiles (2.4%) likely reflects instances where coagulation testing was unavailable in peripheral hospitals, leading to transfusions for stabilisation before transfer to referral centres. The use of de‐identified data prevents verification of these cases, which may affect the accuracy of data linkage in such specific scenarios.

This study has several limitations. The probabilistic nature of data linkage introduces the potential for inaccuracies, including false positives (erroneous links) and false negatives (missed links) [26]. The use of de‐identified data prevents verification against patient medical records, making it difficult to determine whether unlinked records are due to clinical practices or limitations in the linkage process [5]. Some ED presentations resulted in multiple admissions; by focusing on the admission with the longest stay, likely reflecting the definitive care phase, shorter admissions and their clinical details were omitted. Additionally, AUSLAB records the dispatch time of blood products rather than the actual administration time, which may impact analyses requiring precise timing. This study did not include linked medication data or adjust for anticoagulant use. Patients may be on anticoagulants at home, contributing to bleeding, but this is often not captured in hospital coding. Accurate identification would require chart reviews, which were beyond the scope of this dataset.

In the digital era of decompensated cirrhosis, technologies like telemedicine, apps, remote monitoring and risk prediction models have been explored but remain largely non‐ED‐focused [27, 28, 29, 30]. Our study focuses on applied haematology in cirrhosis when patients present to the ED. QH comprises hospitals using both digital and non‐digital platforms, making research across both systems challenging. Our study offers a scalable solution to integrate data from both digital and non‐digital hospitals until full digitalisation of QH is achieved. This approach enables a comprehensive analysis of blood component use in CLD patients across metropolitan, regional, rural and remote areas. It facilitates benchmarking of ED and inpatient blood product utilisation, supporting interstate and national comparisons as well as statewide transfusion data initiatives. Future research using this dataset will evaluate the association between coagulation abnormalities, blood component transfusions and MT practices. This work will also examine viscoelastic testing versus standard coagulation assessments to better understand their role in guiding transfusion decisions.

## 5 Conclusions

This proof‐of‐concept study demonstrates the feasibility of integrating healthcare datasets to evaluate coagulation abnormalities and transfusion practices in patients with CLD presenting to the ED, addressing statewide challenges in applied haematology. By linking ED, admission, pathology and transfusion data, it establishes a scalable model for haemovigilance studies and sets the stage for future research into transfusion practices and outcomes in CLD and broader patient populations.

## Author Contributions

**Akmez Latona**: conceptualisation, methodology, validation, formal analysis, investigation, resources, writing – original draft, visualisation, project administration, funding acquisition. **Emma Smith**: validation, formal analysis, investigation, resources, data curation, writing – reviewing and editing, visualisation. **James Grant**: writing – review and editing. **Biswadev Mitra**: writing – review and editing, supervision

## Consent

A waiver of individual patient consent was granted by the ethics committee due to the use of a large dataset consisting of de‐identified patient information.

## Conflicts of Interest

The authors declare no conflicts of interest.

## Supporting information




**Supporting Table 1**: jha270101‐sup‐0001‐Table_1.docx

## Data Availability

The data is not publicly available due to ethical restrictions. Access is limited to approved investigators under conditions set by the ethics committee. However, researchers interested in collaboration in this under‐researched area of transfusion are encouraged to get in touch. Collaborative projects can be pursued through appropriate ethics applications and governance pathways. For collaboration enquiries, please contact the corresponding author at akmez.latona@health.qld.gov.au.
