# Supplementary material for: Coagulation and Transfusion Informatics in Chronic Liver Disease: A Data Linkage Study of Emergency Department Presentations
Source: EJHaem. 2025 Jul 10;6(4):e70101. doi: 10.1002/jha2.70101 (PMC12244256; doi:10.1002/jha2.70101)
Supplement: Supplementary file 1 — Supporting Table 1: jha270101‐sup‐0001‐Table_1.docx [file JHA2-6-e70101-s001.docx]

**Appendix 1**

**Initial Cohort of patients identified by Data Linkage Queensland in the Master Linkage File and Central Linkage File:**

Adult patients presenting at public emergency departments or admitted to public hospitals, from 1 January 2021 to 31 August 2023, with a principal other diagnosis related to liver disease (Identified by in-scope ICD codes for any type of liver disease).

Adult patients who presented to public emergency departments or were admitted to public hospitals between 1 January 2021 and 31 August 2023, with a principal or additional diagnosis in the Emergency Data Collection (EDC) and/or a primary or other diagnosis in the Queensland Health Admitted Patient Data Collection (QHADPC) related to liver disease (identified by in-scope ICD codes for any type of liver disease).

**In-scope ICD codes used by Data Linkage Queensland:**

| **CODE** | **DESCRIPTION** |
| --- | --- |
| 1850 | Oesophageal varices with bleeding |
| I983 | Oesophageal varices with bleeding in diseases classified elsewhere |
| A064 | Amoebic liver abscess |
| B150 | Hepatitis A with hepatic coma |
| B159 | Hepatitis A without hepatic coma |
| B160 | Acute hepatitis B with delta-agent (coinfection) with hepatic coma |
| B161 | Acute hepatitis B with delta-agent (coinfection) without hepatic coma |
| B162 | Acute hepatitis B without delta-agent with hepatic coma |
| B169 | Acute hepatitis B without delta-agent and without hepatic coma |
| B170 | Acute delta-(super)infection in chronic hepatitis B |
| B171 | Acute hepatitis C |
| B172 | Acute hepatitis E |
| B178 | Other specified acute viral hepatitis |
| B179 | Acute viral hepatitis, unspecified |
| B180 | Chronic viral hepatitis B with delta-agent |
| B181 | Chronic viral hepatitis B without delta-agent |
| 8182 | Chronic viral hepatitis C |
| B188 | Other chronic viral hepatitis |
| B189 | Chronic viral hepatitis, unspecified |
| B190 | Unspecified viral hepatitis with hepatic coma |
| B199 | Unspecified viral hepatitis without hepatic coma |
| B670 | Echinococcus granulosus infection of liver |
| B675 | Echinococcus multilocularis infection of liver |
| B678 | Echinococcosis, unspecified, of liver |
| B251 | Cytomegaloviral hepatitis |
| B581 | Toxoplasma hepatitis |
| B942 | Sequelae of viral hepatitis |
| C183 | Malignant neoplasm of hepatic flexure |
| C220 | Liver cell carcinoma |
| C221 | Intrahepatic bile duct carcinoma |
| C223 | Angiosarcoma of liver |
| C224 | Other sarcomas of liver |
| C227 | Other specified carcinomas of liver |
| C229 | Malignant neoplasm of liver, unspecified |
| C787 | Secondary malignant neoplasm of liver and intrahepatic bile duct |
| D015 | Carcinoma in situ of liver, gallbladder and bile ducts |
| D134 | Benign neoplasm of liver |
| D376 | Neoplasm of uncertain or unknown behaviour of liver, gallbladder and bile ducts |
| K700 | Alcoholic fatty liver |
| K701 | Alcoholic hepatitis |
| K702 | Alcoholic fibrosis and sclerosis of liver |
| K703 | Alcoholic cirrhosis of liver |
| K704 | Alcoholic hepatic failure |
| K709 | Alcoholic liver disease, unspecified |
| K710 | Toxic liver disease with cholestasis |
| K711 | Toxic liver disease with hepatic necrosis |
| K712 | Toxic liver disease with acute hepatitis |
| K713 | Toxic liver disease with chronic persistent hepatitis |
| K714 | Toxic liver disease with chronic lobular hepatitis |
| K715 | Toxic liver disease with chronic active hepatitis |
| K716 | Toxic liver disease with hepatitis, not elsewhere classified |
| K717 | Toxic liver disease with fibrosis and cirrhosis of liver |
| K718 | Toxic liver disease with other disorders of liver |
| K719 | Toxic liver disease, unspecified |
| K720 | Acute and subacute hepatic failure |
| K721 | Chronic hepatic failure |
| K729 | Hepatic failure, unspecified |
| K730 | Chronic persistent hepatitis, not elsewhere classified |
| K731 | Chronic lobular hepatitis, not elsewhere classified |
| K732 | Chronic active hepatitis, not elsewhere classified |
| K738 | Other chronic hepatitis, not elsewhere classified |
| K739 | Chronic hepatitis, unspecified |
| K740 | Hepatic fibrosis |
| K741 | Hepatic sclerosis |
| K742 | Hepatic fibrosis with hepatic sclerosis |
| K743 | Primary biliary cirrhosis |
| K744 | Secondary biliary cirrhosis |
| K745 | Biliary cirrhosis, unspecified |
| K746 | Other and unspecified cirrhosis of liver |
| K750 | Abscess of liver |
| K751 | Phlebitis of portal vein |
| K752 | Nonspecific reactive hepatitis |
| K753 | Granulomatous hepatitis, not elsewhere classified |
| K754 | Autoimmune hepatitis |
| K758 | Other specified inflammatory liver diseases |
| K759 | Inflammatory liver disease, unspecified |
| K760 | Fatty (change of) liver, not elsewhere classified |
| K761 | Chronic passive congestion of liver |
| K762 | Central haemorrhagic necrosis of liver |
| K763 | Infarction of liver |
| K764 | Peliosis hepatis |
| K765 | Hepatic veno-occlusive disease |
| K766 | Portal hypertension |
| K767 | Hepatorenal syndrome |
| K768 | Other specified diseases of liver |
| K769 | Liver disease, unspecified |
| K770 | Liver disorders in infectious and parasitic diseases classified elsewhere |
| K778 | Liver disorders in other diseases classified elsewhere |
| OK9165 | Accidental puncture and laceration of liver during a procedure |
| 0265 | Maternal hypotension syndrome |
| PI 50 | Birth trauma to liver |
| Q446 | Cystic disease of liver |
| OQ4471 | Alagille syndrome |
| OQ4472 | Congenital absence of liver |
| OQ4479 | Other congenital malformations of liver |
| R18 | Ascites |
| R932 | Abnormal findings on diagnostic imaging of liver and biliary tract |
| R945 | Abnormal results of liver function studies |
| OS3610 | Injury of liver, unspecified |
| OS3611 | Contusion and haematoma of liver |
| OS3612 | Laceration of liver, unspecified |
| OS3613 | Minor laceration of liver |
| OS3614 | Moderate laceration of liver |
| OS3615 | Major laceration of liver |
| OS3616 | Other injury of liver |
| T864 | Liver transplant failure and rejection |
| Z944 | Liver transplant status |
| OY8306 | Liver transplant as the cause of abnormal reaction, or of later complication, without mention of unintentional events at the time of the procedure |
| U843 | Chronic liver failure |

**Final cohort of patients with CLD identified in Final Linkage File:**

List of ICD-10AM (ICD-codes) for inclusion in EDC and QHADPC: gastroesophageal varices with/without bleeding (I850, I859, I864, I983, I982), alcohol related liver disease (K700, K701, K702, K703, K704, K709),  toxic liver disease with fibrosis and cirrhosis of liver (K717), hepatic failure (K721, K729), chronic hepatitis (K730, 731, 732, 738, 739), fibrosis and cirrhosis of liver (K740, K741, K743, K744, K745, K746),  other inflammatory liver diseases (K752, K754, K758, K759), K760 (Fatty change of liver, not elsewhere classified, other diseases of liver  (K766, K768, K769),  and chronic viral hepatitis (B18). We also included ascites (R18) in EDC database if it was associated with any of the above codes in QHADPC and/or if it was associated with any of cirrhosis related procedure codes (3047603, 3047602, 9033400, 3040600).
